# Skin-to-Skin Contact in Cesarean Birth and Duration of Breastfeeding: A Cohort Study

**DOI:** 10.1155/2017/1940756

**Published:** 2017-09-07

**Authors:** Andrea Guala, Luigina Boscardini, Raffaella Visentin, Paola Angellotti, Laura Grugni, Michelangelo Barbaglia, Elise Chapin, Eleonora Castelli, Enrico Finale

**Affiliations:** ^1^Department of Pediatrics, Castelli Hospital, Verbania, Italy; ^2^Department of Anesthesia and Resuscitation, Castelli Hospital, Verbania, Italy; ^3^SS Trinità Hospital, Borgomanero, Italy; ^4^Baby Friendly Initiatives, Italian Committee UNICEF, Rome, Italy; ^5^Department of Obstetrics and Gynecology, Castelli Hospital, Verbania, Italy

## Abstract

Early skin-to-skin contact (SSC) after birth is a physiological practice that is internationally recommended and has well-documented importance for the baby and for the mother. This study aims to examine SSC with a cohort of mothers or fathers in the operating room after a Cesarean section (C-section) and its relationship with duration of breastfeeding. From January 1, 2012, to December 31, 2012, at the Castelli Hospital in Verbania, Italy, a Baby Friendly designated hospital, 252 consecutive women who had a C-section were enrolled in the study and followed for 6 months. The sample was later divided into three groups depending on the real outcomes in the operating room: SSC with the mother (57.5%), SSC with the father (17.5%), and no SSC (25%). Our study showed a statistical association between skin-to-skin contact with the mother and the exclusive breastfeeding rates on discharge. This effect is maintained and statistically significant at three and six months, as compared to the groups that had paternal SSC or no SSC. After a C-section, skin-to-skin contact with the mother can be an important practice for support, promotion, and duration of breastfeeding.

## 1. Introduction

Early skin-to-skin contact (SSC) after birth is a physiological practice that is internationally recommended [1–3] and has well-documented importance for the baby and for the mother [4]: prevention of hypothermia [5], decreased crying time, neonatal bonding, cardiorespiratory stability, and early latching on to the breast which leads to breastfeeding [5–8]. Babies born by Cesarean section do not acquire maternal vaginal microbes; thus SSC after birth permits microbial colonization of the newborn with maternal skin microbiota [9]. Significant effects for the mother include the reduction of depressive symptoms and physiological stress during the postnatal period and the strengthening of the mother-baby relationship [10, 11].

SSC is an integral part of the World Health Organization (WHO) and UNICEF's Baby Friendly Hospital Initiative [3] (BFHI) and a practice that requires minimal organizational effort or costs for the hospitals that offer it. SSC has also been shown to have important health impacts [12] and to save money in neonatal units [13]. While SSC should be the natural conclusion after a vaginal birth, it is not always feasible during a Cesarean section (C-section), especially an emergency one [5, 6]. If the mother is unable to do SSC for medical or personal reasons, the father remains an alternative to decrease crying time [14, 15]. This study aims to examine skin-to-skin contact with mother or father in the operating room in the case of a C-section and its relationship to the onset and duration of breastfeeding.

## 2. Materials and Methods

From January 1, 2012, to December 31, 2012, at the Castelli Hospital in Verbania (a Baby Friendly Hospital designated since 2010), 252 women who had a C-section were enrolled in the study. Sample sizes were calculated with a significance level of 95% and a power of 80%. According to Kelsey [16], in order to have a statistically significant sample, the sample size needs to be at least 60 subjects (30 in the control group and 30 in the intervention group), and our sample exceeds the minimum. The inclusion criteria were pregnancy > 37 weeks; APGAR at 5 minutes > 7; any indication for C-section. To reduce confounding factors, the researchers included only women who received information about breastfeeding during pregnancy, as required by the BFHI policy, and that were motivated to breastfeed in the sample. Even partners of women who decided to participate in the study expressed support and motivation for breastfeeding. The entire sample, then, were enrolled only in pairs (pregnant women and partners), were informed and motivated about breastfeeding, and had a C-section at term (>37 weeks). The sample was divided into 3 separate groups, based on the women's choices: SSC with the mother (SSCM), SSC with the father (SSCF), or no SSC (NM_NF SSC).

At the end of their pregnancies, all women who planned to give birth at the Castelli Hospital were told about the possibility of having SSC with their baby, even when a C-section was planned, and they were asked to sign an informed consent form. The possibility and importance of SSC with the newborn were also discussed with the fathers, if SSC with the mother was not possible or not desired.

To ensure SSC in the operating room, our procedure states that, after extraction, the newborn is entrusted to the pediatrician and the nurse for drying off and clinical assessment at 1 and 5 minutes after birth. If the assessment is positive (APGAR > 7), the nurse begins SSC with the mother who is still lying on the operating table. SSC continues throughout the surgical intervention and after the mother returns to the ward for at least 2 hours after birth. If the baby is still breastfeeding (effective latch and suckling) after 2 hours, SSC can last up to three hours.

If the woman does not want SSC in the operating room (OR) or is unable to the father can do it.

Immediately after the pediatrician's clinical evaluation of the infant, he/she is placed skin-to-skin contact with the father in a room adjacent to the operating room during the surgery and while the mother is transferred from the operating room to her hospital room. Once the mother is in the ward, SSC is continued by the mother until at least 2 hours after birth.

In cases where SSC is not done during the C-section, the baby is transported to the neonatal unit and heated with a radiant lamp until the end of surgery and the transfer of the mother to the ward. After that, SSC is done by the mother for at least 2 hours after birth.

During the hospital stay, regardless of SSC mode (mother, father, or any SSC), the BFHI requires us to ensure at least 8 feedings in 24 hours. If the mother and baby need to be separated, hand expression or pumping is encouraged. The milk collected will be given to babies who are unable to feed at the breast, thereby avoiding routine blood sugar testing. In fact, our procedure is to routinely test only in the presence of risk factors (maternal gestational diabetes, use of hypoglycemic agents) or if clinically indicated (gestational age < 37 weeks, IUGR/SGA, fetal distress, birth weight > 4000 gr, hypothermia, polycythemia, suspected sepsis, fetal erythroblastosis, and suspected genetic syndromes).

The study included three rounds of data collection: the first one was carried out by a pediatrician at hospital discharge. The second and third rounds were carried out through telephone interviews at three and six months after delivery (range ± 10 days) by an interviewer who was blind to the group the mothers had been assigned to. The interview questions investigated the baby's eating habits and particularly what had been consumed in the 24 hours preceding the call. All participants completed the entire series of 3 interviews.

The statistical analysis was carried out using OpenEpi, Open Source Epidemiologic Statistics for Public Health software. The statistical tests used included contingency tables and the Kelsey [16] test for the calculation of the sample size.

## 3. Results

All the eligible women expressed a desire to have SSC with their newborns and signed the informed consent form. The sample of 252 women was later divided into three groups depending on the real outcomes in the operating room: SSCM (*n*. 145 pairs; 57.5%), SSCF (*n*. 44; 17,5%), and NM_NF SSC (*n*. 63; 25%). All (100%) cases in which the father did SSC were related to the onset of maternal nausea/discomfort in the immediate postpartum. Of those newborns who had no SSC, most did not because of maternal complications from anesthesia (39.5%), closely followed by paternal refusal (34.8%).

The breakdown of reasons for a C-section is shown in Table 1.

The statistical analysis examined only the exclusive breastfeeding as a discriminating factor.

The comparisons between percentages of exclusive breastfeeding of three groups (SSCM, SSCF, and NM_NF SSC) were carried out three times that were taken into account (T0, 3 months, and 6 months).

The differences inside the three groups have been evaluated by chi2 (global comparison). Moreover a valuation was conducted about the presence of a linear trend. The differences between proportions were quantified with a two-sample test for proportions, calculating their relatives confidence intervals (not corrected for the multiple comparisons).

The first analysis showed interesting data regarding the feeding babies on discharge (T0) and studied the differences between the three groups of exclusive breastfeeding: overall the existence of a significant statistic has been demonstrated (*p* < 0.0001) between babies that did the skin-to-skin contact with their mothers and babies that did the skin-to-skin contact with their father and no skin-to-skin contact. Also the crossing of the data of the individual proportions of groups (SSCM versus SSCF; M versus NM_NF SSC) confirms a significant statistic.

The second analysis has taken into account the relatives data at the first follow-up (three months after discharge), and the linear trend has been maintained at this stage: it seems that the skin-to-skin contact with mother represents a support factor of the exclusive breastfeeding. The crossing of the data of the individual proportions of the groups showed a more pronounced statistical difference between the skin-to-skin contact group with the mother and the group that did not perform skin-to-skin contact (*p* 0.001 versus* p*  0.0084).

The third analysis has taken into consideration the second follow-up (six months after discharge).

**Figure 1:**
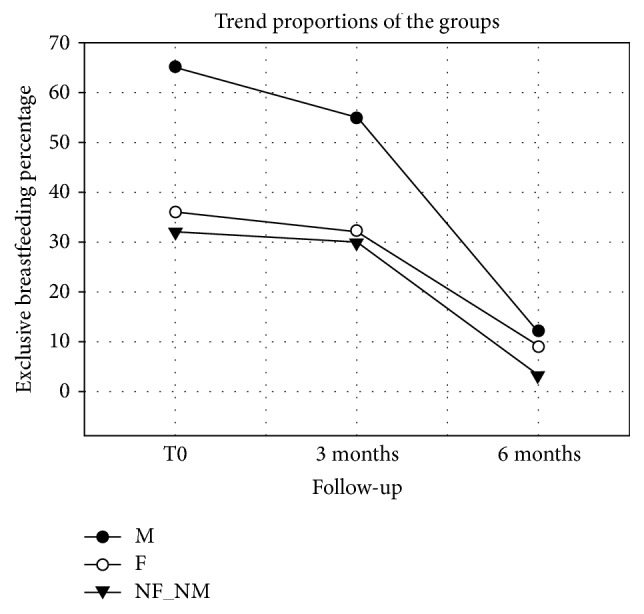
Linear regression of the three groups. M: SSC mother; F: SSC father; NM_NF: no SSC.

The significant statistic is less marked compared to the previous analyzes, and the linear trend seems to be less pronounced for the small sample size. Although proportionally having skin-to-skin contact with the mother appears to be associated with an exclusive breastfeeding period longer than the other groups as can be seen in Figure 1.

Table 2 shows the complete statistical analysis for the three reference periods.

## 4. Conclusion

C-section typically reduces breastfeeding [17] and our experience confirms that babies born by C-section have a lower rate of breastfeeding at hospital discharge compared to those born by vaginal delivery.  Table 3 shows the rates of any breastfeeding at hospital discharge, as well as the differences between the C-section group (93.2%) and the vaginal one (99.4%) in our Baby Friendly Hospital.

Our study showed a statistical association between skin-to-skin contact with the mother and the exclusive breastfeeding rates for discharge. This effect is still maintained at three and six months. As recommended by the BFHI, SSC with the mother after a C-section should be done as often as possible since our data show, with a statistical association, an improvement in the rate of exclusive as compared to the group that did not do any skin-to-skin contact in the operating room or that did it with the father.

The neuroendocrine mechanisms of the genesis of lactation recognize that mother-to-newborn is the most effective and powerful stimulus to milk production, in addition, of course, to the mother's motivation that to date is the most important factor for the breastfeeding's success.

Thus, SSC with the father after C-section did not affect breastfeeding rates positively, or rather the results are similar to the group that did no SSC in the operating room. Although there may be no beneficial impact on breastfeeding, we hypothesize, however, that if the mother is unwilling or unable to do SSC, this practice should still be encouraged in order to give fathers an opportunity to bond with their newborns [14, 15] and to colonize the newborn with familial bacteria, even if further studies are needed to confirm this hypothesis.

The only statistical association is not enough to declare a causal association. Probably the women who made skin-to-skin contact in the operating room were the women most likely to breastfeed, but obviously the practice of skin-to-skin care in the operating room for those born by C-section should be integrated with all the other best practices that have proven effective in promoting breastfeeding [18, 19] and that we practice as a Baby Friendly Hospital every day.

A limit of our study was the fact that only women who had received BFHI-compatible information were included. It is possible that these results cannot be generalized to a population that does not have the same characteristics as ours.

## Figures and Tables

**Table 1:** Indication for Cesarean section.

Indication for Cesarean section	*n*
Podalic presentation	11

Previous C-section in a woman who has refused VBAC	125

Maternal medical indications (cardiological, orthopedic, psychiatric, infectivological)	71

Multiple pregnancy with one of the fetuses presenting podalically	7

Placenta praevia	6

Fetal distress	27

Placental abruption	2

Labor in patient with placenta praevia	1

Labor in patient with podalic presentation	2

**Table 2:** Complete statistical analysis.

Groups^†^	T0			3M			6M		
	*n*/tot	%	95% CI	*n*/tot	%	95% CI	*n*/tot	%	95% CI
M	95/145	65	(57–73)	79/145	55	(46–63)	18/145	12	(7.5–19)
F	16/44	36	(22–52)	14/44	32	(19–48)	4/44	9	(2.5–22)
NM_NF	20/63	32	(21–45)	19/63	30	(19–43)	2/63	3	(0.4–11)

Overall *p* Chi2	<0.0001			0.0009			0.1129		
*p* for trend Chi2	<0.0001			0.0004			0.0383		

*Difference between proportions (%)*									
Contrasts	*p* ^‡^	95% CI diff		*p* ^‡^	95% CI diff		*p* ^‡^	95% CI diff	

M versus F	0.0006	(13–45)		0.0084	(6.7–39)		0.547	(6.7–13)	
M versus NM_NF	<0.0001	(20–48)		0.001	(10–38)		0.0378	(2.3–16)	

^†^M: SSC mother; F: SSC father; NM_NF: no SSC; ^‡^*p* values uncorrected for multiple comparisons; T0: discharge; 3M: 3 months; 6M: 6 months.

**Table 3:** Percentage feeding type at discharge.

Vaginal deliveries versus C-section			
*n*		Any breastfeeding	Formula
Vaginal birth	327	325 (99,4%)	2
C-section	252	235 (93,2%)	17

## Abbreviations

SSC: Skin-to-skin
SSCM: Skin-to-skin with mother
SSCF: Skin-to-skin with father
NM_NF SSC: No mother, no father skin-to-skin
C-section: Cesarean section.
